# Actions of Hydrogen Sulfide and ATP-Sensitive Potassium Channels on Colonic Hypermotility in a Rat Model of Chronic Stress

**DOI:** 10.1371/journal.pone.0055853

**Published:** 2013-02-06

**Authors:** Ying Liu, Hesheng Luo, Chengbo Liang, Hong Xia, Wenjuan Xu, Jihong Chen, Mingkai Chen

**Affiliations:** 1 Department of Gastroenterology, Renmin Hospital of Wuhan University, Wuhan, China; 2 Department of Gastroenterology, Affiliated Hospital of Guilin Medical College, Guilin, China; University of California, Los Angeles, United States of America

## Abstract

**Objective:**

To investigate the potential role of hydrogen sulphide (H_2_S) and ATP-sensitive potassium (K_ATP_) channels in chronic stress-induced colonic hypermotility.

**Methods:**

Male Wistar rats were submitted daily to 1 h of water avoidance stress (WAS) or sham WAS (SWAS) for 10 consecutive days. Organ bath recordings, H_2_S production, immunohistochemistry and western blotting were performed on rat colonic samples to investigate the role of endogenous H_2_S in repeated WAS-induced hypermotility. Organ bath recordings and western blotting were used to detect the role of K_ATP_ channels in repeated WAS.

**Results:**

Repeated WAS increased the number of fecal pellets per hour and the area under the curve of the spontaneous contractions of colonic strips, and decreased the endogenous production of H_2_S and the expression of H_2_S-producing enzymes in the colon devoid of mucosa and submucosa. Inhibitors of H_2_S-producing enzymes increased the contractile activity of colonic strips in the SWAS rats. NaHS concentration-dependently inhibited the spontaneous contractions of the strips and the NaHS IC_50_ for the WAS rats was significantly lower than that for the SWAS rats. The inhibitory effect of NaHS was significantly reduced by glybenclamide. Repeated WAS treatment resulted in up-regulation of Kir6.1 and SUR2B of K_ATP_ channels in the colon devoid of mucosa and submucosa.

**Conclusion:**

The colonic hypermotility induced by repeated WAS may be associated with the decreased production of endogenous H_2_S. The increased expression of the subunits of K_ATP_ channels in colonic smooth muscle cells may be a defensive response to repeated WAS. H_2_S donor may have potential clinical utility in treating chronic stress- induced colonic hypermotility.

## Introduction

Different psychological and environmental stressors affect physiologic functions of the gastrointestinal tract and play important roles in the pathophysiology of gastrointestinal diseases [1]. Chronic stress causes colonic hypermotility [2], [3], [4], [5], [6] and precipitates or exacerbates the symptoms of two major motility disorders, irritable bowel syndrome and inammatory bowel disease [4], [7]. The mechanisms that underline this increased colonic motility has received increased awareness in the past years. Experimental studies have revealed that some factors are involved, such as central nervous system,brain-gut axis, neurotransmitters, gastrointestinal hormones, and L-type Ca^2+^ channels located in the colon [2], [4], [5], [6], [7], [8].

Hydrogen sulfide (H_2_S) has recently been identified as a new “gasotransmitter”. It is synthesized in many mammalian tissues and produces effects on various biological targets that have widespread consequences, ranging from cytotoxic to cytoprotective [9]. Cystathionine β-synthase (CBS) and systathionine γ-lyase (CSE) are two important enzymes for generation of endogenous H_2_S [9]. They have been shown to be expressed in the smooth muscle cells, enteric neurons, interstitial cells of Cajal, and epithelial cells of the gastrointestinal (GI) tract [10]. There is growing evidence that endogenous H_2_S might play an important role in several physiological processes including neurotransmission, pain, motility, and secretion [10], [11], [12]. Pharmacological studies show that exogenously applied NaHS, a H_2_S donor, inhibits gastric and intestinal motility, causing GI smooth muscle relaxation [13], [14], [15], [16], [17].

The mechanism through which H_2_S exerts its relaxant properties is related to the direct opening of ATP-sensitive potassium (K_ATP_) channels located in the smooth muscle cells [9], [10], [14], [15], [18]. Other potential targets of action of H_2_S on GI smooth muscle include apamin-sensitive SK channels and delayed rectifier potassium channels [14], [15]. K_ATP_ channels are composed of at least two subunits: an inwardly rectifying K^+^ channel six family (Kir6.x) that forms the ion conducting pore and a modulatory sulphonylurea receptor (SUR) that accounts for several pharmacological properties [19], [20]. Both Kir and SUR subunits must be co-expressed, and combine in a 4∶4 stoichiometry to generate a functional K_ATP_ channel [19], [20]. It is now well recognized that K_ATP_ channels locate in GI smooth muscle cells, and Kir 6.1/SUR2B and Kir 6.2/SUR2B form the K_ATP_ complex [21], [22], [23], [24]. Differences exist in the functional and pharmacological properties of various K_ATP_ channels in different tissues. In GI tract, the physiological role of K_ATP_ channels may be related to the modulation of cell excitability [21]. Activation of K_ATP_ channels leads to an increased hyperpolarization of membrane potential and results in the relaxation of GI smooth muscle [10].

Given the role of H_2_S and K_ATP_ channels in GI motility, we investigated the possibility that H_2_S and/or K_ATP_ channels contribute(s) to the colonic motility dysfunction in chronic stress. This involved an investigation of colonic H_2_S synthesis and the expression of two key enzymes for H_2_S synthesis over the course of repeated water avoidance stress (WAS). We also examined whether or not blocking H_2_S synthesis in sham stress could mimic the colonic hypermotility in chronic stress. Finally, we examined the potential role of exogenous H_2_S donor and K_ATP_ channels in chronic WAS.

## Materials and Methods

### Animals

Adult male Wistar rats weighting 200–220 g were obtained from the Experimental Animal Center of Wuhan University, Wuhan, Hubei Province, China. They were kept under conventional conditions in an environmentally controlled room (20–21°C, 60% humidity, 12∶12 h light–dark cycle). All protocols were approved by the Institutional Animal Care and Use Committee of Wuhan University (Approval ID: WHU20110312) and adhered to the ethical guidelines of the International Association for the Study of Pain.

### WAS Protocol

The WAS was conducted following the procedures modified from previous reports [2], [3], [8], [25]. Briefly, the animals were placed on a block (8×6×6cm), which was fixed in the center of a tank for a period of 1 h daily for 10 consecutive days. The tank was filled with fresh tap water up to 1 cm from of the top edge of the block. The sham WAS (SWAS) consisted of placing the rats similarly for 1 h daily for 10 days on the same platform in a waterless container. Stress sessions were performed between 10∶00 AM and 13∶00 PM to minimize diurnal variations in response. The total number of fecal pellets was counted at the end of each 1 h WAS or SWAS session.

### Colonic Motility Tests in vitro

Rats were killed by cervical dislocation 30 min after the last stress session. The proximal colon was carefully removed and opened along the mesenteric border and pinned mucosa side up. The mucosa and submucosa were removed by sharp dissection under a dissection microscope. The circular muscle (CM) or longitudinal muscle (LM) strips (3×10 mm) were cut along the circular or longitudinal axis. Each smooth muscle strip was suspended in an 6 ml organ bath which was filled with Tyrode’s buffer containing(mM): NaCl 147.0, KCl 4.0, CaCl_2_ 2.0, NaH_2_PO_4_ 0.42, Na_2_HPO_4_ 2.0, MgCl_2_ 1.05, and glucose 5.5 (adjusted to pH 7.4 with NaOH). The organ bath was bubbled with a mixture of 95% O_2_ plus 5% CO_2_ and maintained at 37°C. One end of the strip was fixed to a hook on the bottom of the chamber and the other end was connected to a isometric force transducer (JZJOIH, Chengdu, CHN) at the top. Each muscle strip was placed under a resting preload of 1.0 g and equilibrated for 60 min, with Tyrode’s buffer being changed every 20 min. To estimate the contractile activity of the strips, the area under the curve (AUC) of the spontaneous contractions from the baseline was measured and the result was expressed in g·min^−1^. To normalize data, the value of AUC obtained before the treatment was considered 100% and the percentage of inhibition of the spontaneous contractions was estimated with the AUC obtained after the treatment.

### Endogenous Production of Hydrogen Sulphide

Production of H_2_S was measured based on previous reports [26], [27]. Briey, the colonic tissue devoid of mucosa and submucosa was placed in a sealed vial containing physiological saline solution with L-cysteine (10 mM) and pyridoxal 5′-phosphate (2 mM) which was connected to a second vial containing 0.5 mL of 1% (w v^−1^) zinc acetate. The first vial was bubbled with a gas mixture of 95% O_2_ and 5% CO_2_ at a rate of 1–4 mL·min^−1^ in order to minimize the degradation of H_2_S. The increase in pressure in the first vial forced the gases bubbled through the zinc acetate solution in the second vial and H_2_S was trapped as zinc sulphide. The incubation mixture was prepared on ice and the reaction was started by transferring the vials to a water bath at 37°C. The reaction was stopped at 30 min by injecting 0.5 mL of 50% (w v^−1^) trichloroacetic acid. Gas ow was allowed to continue for an additional 30 min to ensure complete trapping of H_2_S.

H_2_S was measured by using a colorimetric method [26], [28]. The content of the second vial was transferred to a test tube and 3.5 mL of distilled water, 0.4 mL of N,N-dimethyl-p-phenylenediamine sulphate (20 mM) in HCl (7.2 M) and 0.4 mL of FeCl_3_ (30 mM) in HCl (1.2 M) were added to the tube. After 20 min of incubation at room temperature, the absorbance of the resulting solution at 670 nm was measured with a spectrophotometer (Hach, Loveland, CO, USA). The calibration curve of absorbante versus sulfide concentration was set up with defined concentrations of NaHS solution and the concentration of H_2_S was estimated and expressed in nM·min^−1^·g^−1^ tissue.

### Immunohistochemistry

The streptavidin–peroxidase method was used for the immunohistological localisation of H_2_S-producing enzymes in proximal colon samples. The tissues were fixed overnight at room temperature in a pH-neutral, phosphatebuffered, 10% formalin solution and then embedded in paraffin. The sections were incubated at 100°C in 10 mM citrate buffer (pH 6.0) for 10 min to retrieve antigens, cooled for 20 min and then washed in phosphate-buffered saline. The sections were cut off, mounted on glass slides and de-paraffinized. Endogenous peroxidase activity was blocked with a 15-min incubation in 3% hydrogen peroxide. Mouse monoclonal anti-CSE and anti-CBS antibodies (1∶250, 1∶300, respectively) were applied to detect CSE and CBS. The sections were incubated in above primary antibodies overnight at 4°C. After washed in PBS, the sections were incubated in biotinylated anti-mouse or anti-goat secondary antibody and streptavidin–horseradish peroxidase. 3,3′-Diaminobenzidine was used as a chromogen and hematoxylin was used for counterstaining.

### Western Blotting

The proximal colon devoid of mucosa and submucosa was homogenized in ice-cold RIPA lysis buffer composed of 20 mM Tris-HCl, 0.1 mM PMSF, and 5ul/ml protease inhibitor cocktail. The protein concentration was determined by Bio-Rad protein assay (Bio-Rad Laboratories, Hercules, CA, USA). Then equal amounts of protein (50 ug) was loaded onto a 10% gel, subjected to SDS-PAGE, and electrotransferred onto polyvinylidene difluoride membranes at a constant current of 200 mA for 95 min at 4°C in a solution containing 25 mM Tris, 193 mM glycine, and 10% methanol. The anti-CSE antibody (1∶1000) and anti-CBS antibody (1∶1500) were applied to detect CSE and CBS. The anti-Kir6.1, anti-Kir6.2 and anti-SUR2B antibodies (1∶400) were used for the detection of Kir6.1, Kir6.2 and SUR2B respectively, with GAPDH (1∶ 10000) as the internal standard. Immune complexes were detected with horseradish peroxidase-conjugated secondary antibodies and enhanced with chemiluminescent substrate. Gradation of the protein bands was analyzed using Bandscan software (Glyko, Novato, USA). The gradation ratio of the target protein immunoreactive band and GAPDH from each lane was calculated as normalized blot optional density.

### Chemicals

NaHS, tetrodotoxin (TTX), glybenclamide, D,L-propargylglycine (PAG) and amino-oxyacetic acid (AOAA) were purchased from Sigma-Aldrich (Sigma-Aldrich Co., St. Louis, MO, USA). The CBS and CSE monoclonal antibodies were purchased from ABNOVA (ABNOVA, Taipei, Taiwan), the anti-Kir6.1, anti-Kir6.2 and anti-SUR2B polyclonal antibodies from Santa Cruz Biotechnology (Santa Cruz Biotechnology, Santa Cruz, CA, USA). GAPDH and streptavidin–horseradish peroxidase were purchased from Zhongshan Goldenbridge Biotechnology (Zhongshan Goldenbridge Biotechnology, Beijing, CHN). All chemicals were dissolved in distilled water except for glibenclamide which was dissolved in DMSO.

### Data Analysis and Statistics

Data were expressed as mean ± SEM. Significant difference between groups was evaluated using unpaired or paired Student’s t-tests. IC_50_ values (i.e., agonist concentrations that produced 50% inhibition of the observed maximum response, respectively) were analyzed using the ‘sigmoidal dose–response (variable slope)’ option for curve fitting. Statistical analysis was performed with Graph Pad Prism 5.0 (GraphPad Software, San Diego, CA, USA). A *P* value below 0.05 was considered statistically significant.

## Results

### Repeated WAS Stimulated Colonic Transit

**Figure 1:**
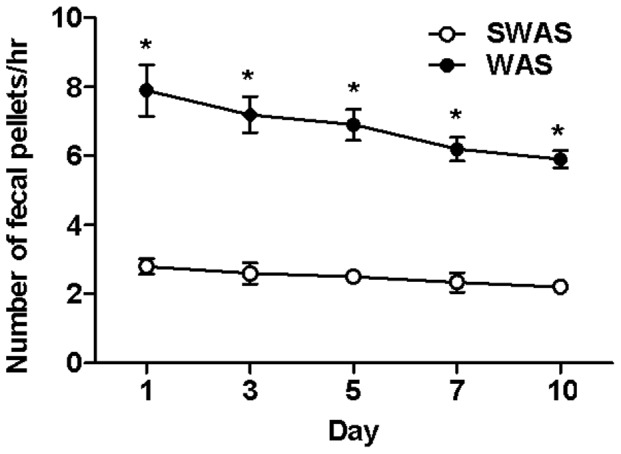
WAS increased number of fecal pellets. WAS increased number of the fecal pellets per hour at days 1, 3, 5, 7 and 10. Data are expressed as mean ± SEM (*n* = 10/group). Significant difference is indicated by *(*P*<0.001 compared to the SWAS rats, according to the unpaired Student’s t-test).

The colonic transits were measured by fecal pellet output. The fecal pellets per hour in the WAS rats at days 1, 3, 5, 7 and 10 were significantly higher than those in the SWAS rats (7.9±0.74 vs. 2.8±0.22, 7.2±0.52 vs. 2.6±0.30, 6.9±0.45 vs. 2.5±0.16, 6.2±0.35 vs. 2.34±0.28 and 5.9±0.25 vs. 2.2±0.12, respectively, *n* = 10/group, *P*<0.001), ever though it had decreased by ∼28% from day 1 (Figure 1).

### Repeated WAS Increased Contractile Activity of Colonic Strips

**Figure 2:**
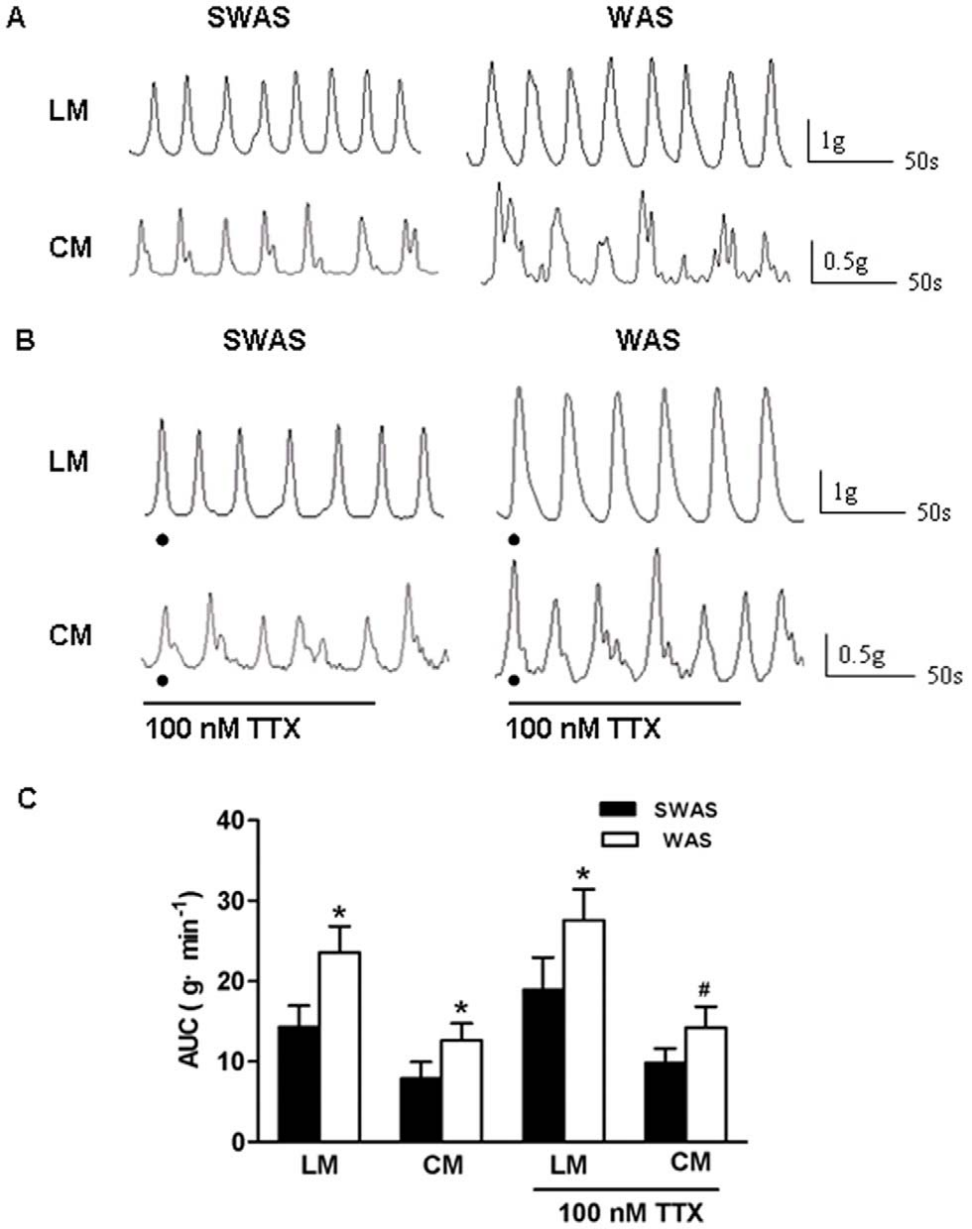
Repeated WAS increased contractile activity of colonic strips. A, B: Original traces of the effect of SWAS or WAS on the spontaneous contractile activity of longitudinal muscle (LM) and circular muscle (CM) in the absence (A) and presence (B) of TTX (100 nM). C: Summarized results showing that repeated WAS increased the spontaneous contractile activity of CM and LM strips in the absence and presence of TTX. Data are expressed as mean ± SEM (*n* = 10/group). Significant difference between the WAS and SWAS rats was evaluated by using the unpaired Student’s t-test. *P<0.001, ^#^ *P*<0.01 compared to the SWAS rats.

The AUC of LM and CM strips of the WAS rats were both significantly higher than those of the SWAS rats (23.59±3.23 g·min^−1^ vs. 14.38±2.56 g·min^−1^ and 12.62±2.13 g·min^−1^ vs. 7.97±1.94 g·min^−1^, *n* = 10/group, *P*<0.001, Figure 2A, 2C). To identify whether alteration in smooth muscle level contributes to the changes of colonic contractile response, colon segments were pretreated with tetrodotoxin (TTX) in organ bath for 30 min in order to block the inuence of the enteric nervous system on smooth muscle contractions. Figure 2B and 2C show that the AUC of LM and CM strips of the WAS rats were still higher than those of the SWAS rats when pretreated with 100 nM TTX (27.58±3.84 g·min^−1^ vs. 19.01±3.95 g·min^−1^, *n* = 10/group, *P*<0.001 and 14.17±2.68 g·min^−1^ vs. 9.89±1.76 g·min^−1^, *n* = 10/group, *P*<0.01).

### H_2_S Synthesis was Decreased Following Repeated WAS

**Figure 3:**
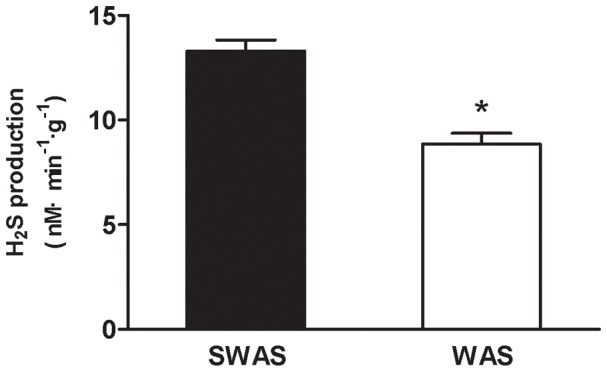
H_2_S synthesis was decreased following repeated WAS. The production of H_2_S in the colonic tissue devoid of mucosa and submucosa was decreased following repeated WAS. Data are expressed as mean ± SEM (*n* = 10/group). Significant difference is indicated by *(*P*<0.001 compared to the SWAS rats, according to the unpaired Student’s t-test).

The level of H_2_S produced by the colonic tissue devoid of mucosa and submucosa in the WAS rats was decreased, as compared with that in the SWAS rats (8.85±1.63 nmol·min^−1^·g^−1^ vs. 13.29±1.72 nmol·min^−1^·g^−1^ tissue, *n* = 10/group, *P*<0.001, Figure 3).

### Repeated WAS Decreased Expression of H_2_S-producing Enzymes

**Figure 4:**
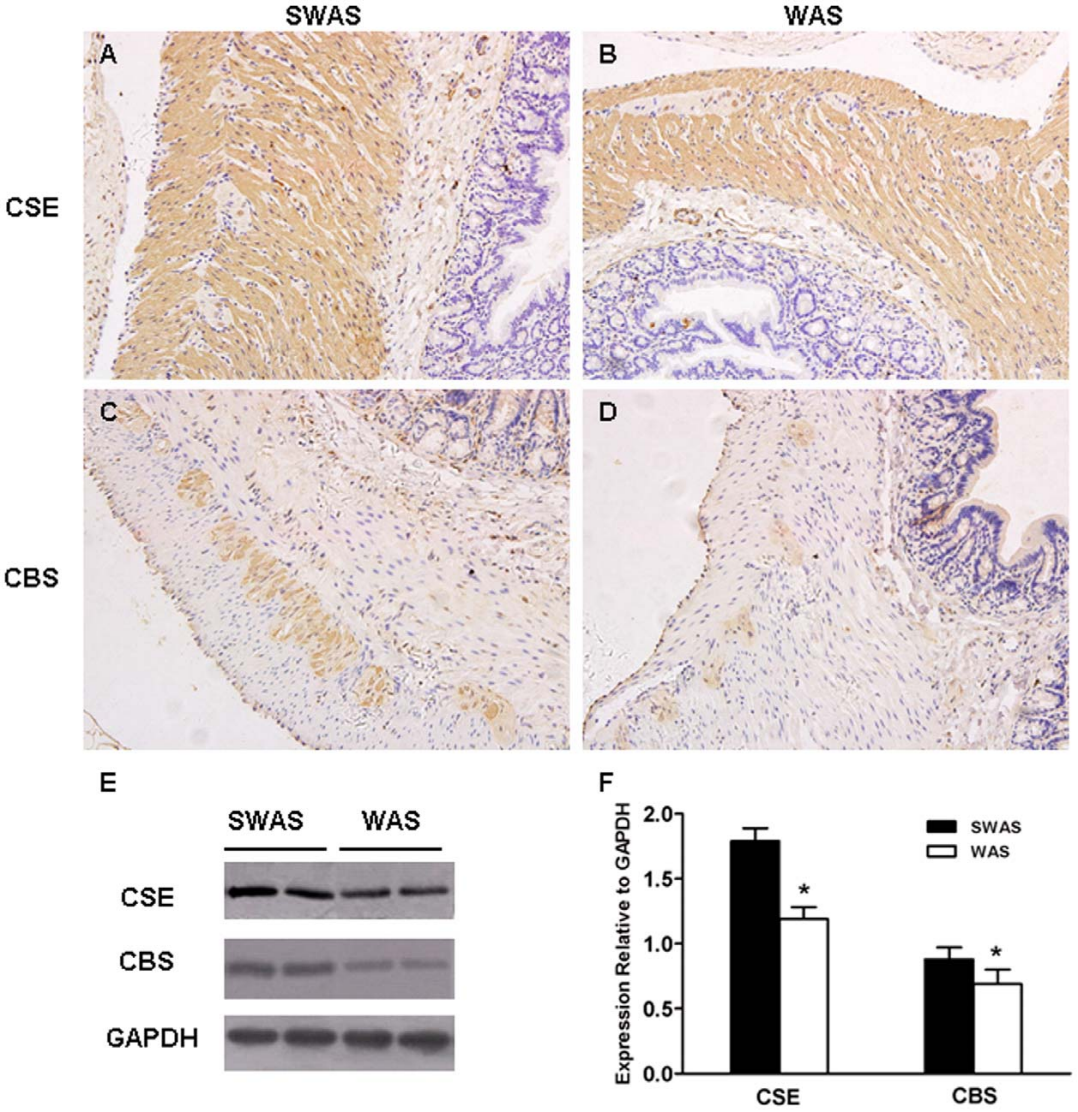
Repeated WAS decreased expression of H_2_S-producing enzymes. A, B: Distribution of CSE in the SWAS (A) and WAS (B) rats colonic tissue. C, D: Distribution of CBS in the SWAS (C) and WAS (D) rats colonic tissue. E: Representative traces of the expression of CSE and CBS in the SWAS and WAS rats. F: Summarized results showing that WAS decreased the expression of H_2_S-producing enzymes. Data are expressed as mean ± SEM (*n* = 10/group). Significant difference between the WAS and SWAS rats was evaluated by using unpaired Student’s t-test. *P<0.001, ^#^ *P*<0.01 compared to the SWAS rats. Magnification×200.

In both SWAS and WAS rats colon, CSE was strongly expressed in the cytosols of the circular and longitudinal smooth muscle cells and the nucleus of the myenteric plexus neurons. CSE-immunoreactivity(IR) was diffuse in the mucosa and submucosa layers (Figure 4A, 4B). A completely different pattern was found for CBS. It was primarily localized in the cytosols of myenteric plexus neurons and weakly localized in the epithelial cells. A diffuse pattern was also observed in the muscular layers (Figure 4C, 4D). Western blotting analysis showed that WAS treatment resulted in down-regulation of the expression of CBS and CSE in the colonic tissue devoid of mucosa and submucosa (*P*<0.001, Figure 4E, 4F).

### Inhibitors of H_2_S-producing Enzymes Increased Contractile Activity of Colonic Strips in SWAS Rats

Since our present experiments demonstrated that the two H_2_S-producing enzymes were both down-regulated in the WAS rats, we determined whether alternatively blocking CSE and CBS using enzyme inhibitors, which mimiced a down regulation of the enzymes, produced an increase in the contractile activity in the SWAS rats.

**Figure 5:**
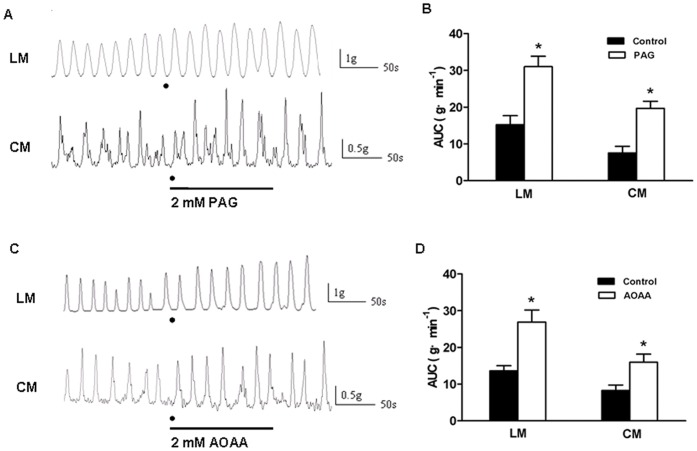
Inhibitors of H_2_S-producing enzymes increased contractile activity of colonic strips in SWAS rats. A, C: Original traces of the spontaneous contractions of longitudinal muscle (LM) and circular muscle (CM) in the SWAS rats after the strips were incubated with PAG (2 mM) or AOAA (2 mM) for 2 min. Both PAG and AOAA increased the contractile activity of colonic strips in SWAS rats. B, D: Summarized results showing that both PAG and AOAA increased the contractile activity of colonic strips in the SWAS rats. Data are expressed as mean ± SEM (*n* = 10/group). Significant difference is indicated by *(*P*<0.001 compared to the controls, according to the paired Student’s t-test).

PAG is an inhibitor of CSE and AOAA, an inhibitor of CBS. After 2 min of incubation with PAG (2 mM) or AOAA (2 mM), the AUC was measured. PAG significantly increased the AUC of LM and CM strips in the SWAS rats (Control: 15.21±2.48 g·min^−1^ vs. PAG: 31.02±2.82 g·min^−1^ and Control: 7.50±1.85 g·min^−1^ vs. PAG: 19.69±1.91 g·min^−1^, *n* = 10, *P*<0.001, Figure 5A, 5B). Similar results were obtained with AOAA (Control: 13.60±1.43 g·min^−1^ vs. AOAA: 26.91±3.26 g·min^−1^ and Control: 8.25±1.50 g·min^−1^ vs. AOAA: 15.95±2.20 g·min^−1^, *n* = 10, *P*<0.001, Figure 5C, 5D).

### NaHS, an Exogenous H_2_S Donor Inhibited Contractile Activity of Colonic Strips

**Figure 6:**
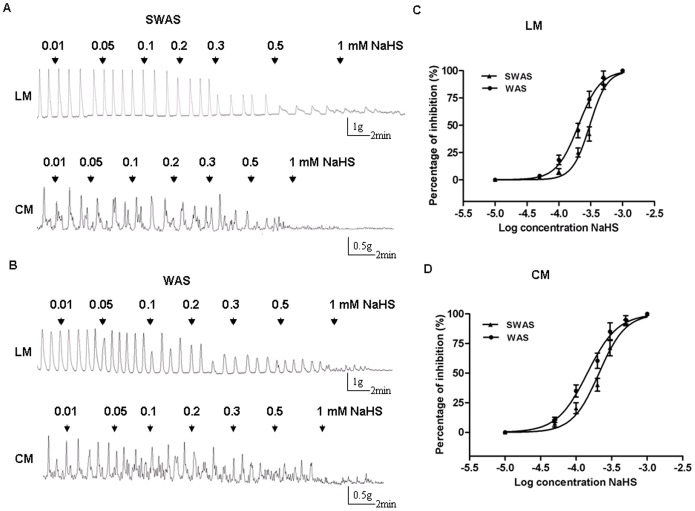
NaHS inhibited contractile activity of colonic strips. A, B: Representative effects of different concentrations of NaHS on the spontaneous contractions of longitudinal muscle (LM) and circular muscle (CM) in the SWAS and WAS rats. C, D: The concentration-dependent curves fitting showing that NaHS inhibited the contractile activity of LM and CM in both SWAS and WAS rats. IC_50_ values of NaHS for the WAS rats were lower than those for the SWAS rats. Data are expressed as mean ± SEM (*n* = 10/group). IC_50_ values were analyzed using iterative curve-fitting methods as described in Section 2.

In both SWAS and WAS rats, NaHS (0.01–1 mM) caused an inhibition of the spontaneous contractions of LM and CM strips (Figure 6A, 6B). This effect was concentration-dependent, with maximal (100%) inhibition occurring at 1 mM. In the LM strips of the WAS rats, the NaHS IC_50_ was 0.2033 mM (95% confidence interval IC_50_ 0.187–0.221, log IC_50_ = −3.692±0.015), significantly lower than that for the SWAS rats (IC_50_ = 0.3054 mM, 95% confidence interval IC_50_ 0.271–0.345, log IC_50_ = −3.515±0.020, *n = *10/group, *P*<0.0001) (Figure 6C). In the CM strips of the WAS rats, the NaHS IC_50_ was 0.1438 mM (95% confidence interval IC50 0.127–0.162, log IC_50_ = −3.842±0.021), also significantly lower than that for the SWAS rats (IC_50_ = 0.210 mM, 95% confidence interval IC_50_ 0.181–0.244, log IC_50_ = −3.678±0.025, *n = *10/group, *P*<0.0001) (Figure 6D).

### Inhibitory Effect of NaHS was Reversed by a K_ATP_ Channel Blocker

**Figure 7:**
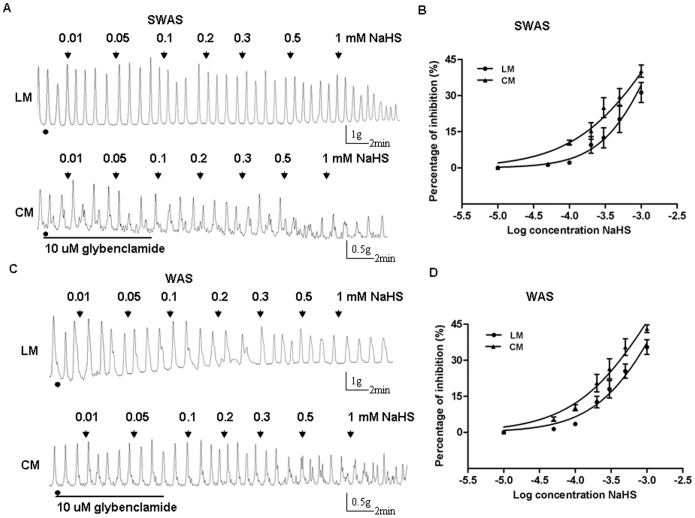
Inhibitory effect of NaHS was reversed by glybenclamide. A, C: Representative effects of different concentrations of NaHS on the spontaneous contractions of longitudinal muscle (LM) and circular muscle (CM) in the SWAS and WAS rats after the strips were incubated with glybenclamide (10 uM) for 30 min. B, D: The concentration-dependent curves fitting showing that glybenclamide significantly reduced the inhibitory effect induced by H_2_S. NaHS (1 mM) only caused 30–45% of inhibition with previous addition of glybenclamide. Data are expressed as mean ± SEM (*n* = 10/group). IC_50_ values were analyzed using iterative curve-fitting methods as described in Section 2.

The inhibitory effect induced by NaHS was significantly reduced after the strips of the two groups were incubated with glybenclamide (10 uM), a K_ATP_ channel blocker for 30 min. NaHS (1 mM) only caused 30–45% of inhibition with previous addition of glybenclamide. In the SWAS rats, glybenclamide increased NaHS IC_50_ from 0.3054 mM to 2.102 mM (95% confidence interval IC_50_ 1.622–2.725, logIC_50_ = −2.677±0.044, *n* = 10/group, *P*<0.0001) for the LM strips and from 0.210 mM to1.484 mM (95% confidence interval IC_50_ 0.991–2.224, logIC_50_ = −2.828±0.068, *n* = 10/group, *P*<0.0001) for the CM strips (Figure 7A, 7B). In the WAS rats, glybenclamide increased NaHS IC50 from 0.2033 mM to 1.756 mM (95% confidence interval IC_50_ 1.235–2.496, logIC_50_ = −2.756±0.059, *n* = 10/group, *P*<0.0001) for the LM strips and from 0.1438 mM to 1.231 mM (95% confidence interval IC_50_ 0.900–1.682, logIC_50_ = −2.910±0.053, *n* = 10/group, *P*<0.0001) for the CM strips (Figure 7C, 7D).

### Repeated WAS Increased Expression of Subunits of K_ATP_ Channels

**Figure 8:**
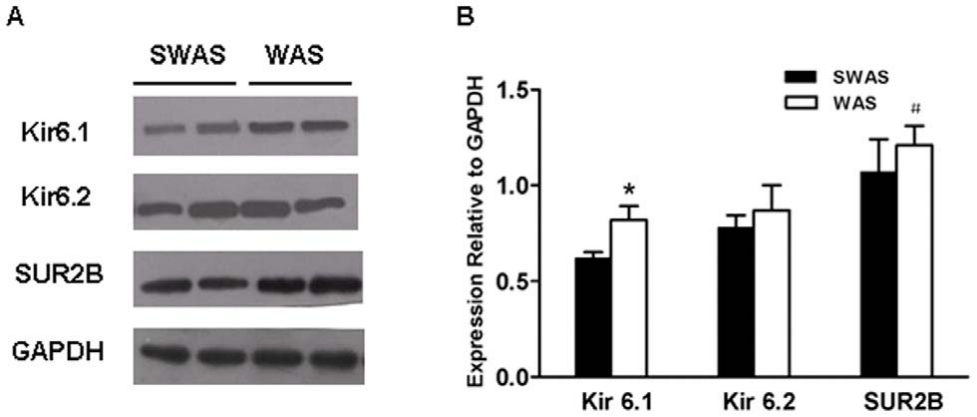
Repeated WAS increased expression of subunits of K_ATP_ channels in colonic tissue devoid of mucosa and submucosa. A: Representative traces of the expression of subunits of K_ATP_ channels in the SWAS and WAS rats. B: Summarized results showing that repeated WAS increased the expression of Kir 6.1 and SUR2B in colonic tissue devoid of mucosa and submucosa. Data are expressed as mean ± SEM (*n* = 10/group). Significant difference between the WAS and SWAS rats was evaluated by using unpaired Student’s t-test. *P<0.001, ^#^ *P*<0.01 compared to the SWAS rats.

Western blotting analysis showed that there was a marked increase in the expression of Kir6.1 and SUR2B, whereas Kir6.2 had no change following WAS (*P*<0.05, Figure 8A, 8B).

## Discussion

The repetitive WAS rat model stimulates the desire to survive as well as fear of the surrounding environment, and is an acceptable animal model for studying the possible mechanisms involved in altered colonic motility and visceral hyperalgesia induced by chronic stress [2], [3]. The present study found that WAS increased the fecal pellet number, a validated measure of colonic motility [2], [3], and the spontaneous contractile activity of colonic strips in the presence or absence of TTX. These data indicate that WAS induces colonic hypermotility, and the increased contractile activity induced by WAS might be directly due to myogenic changes in the colon, even though the potential changes of enteric nervous system and brain-gut axis may have contribution as well.

H_2_S is produced enzymatically by GI tissues and causes well-defined physiologic effects including inhibition of motility and causing smooth muscle relaxation [10], [11], [12]. However, little is known about the link between the hypermotility induced by repeated stress and the endogenous synthesis of H_2_S. We, therefore, examined the possibility that hypermotility induced by repeated stress may have been due to the decrease in H_2_S synthesis. First, we observed the endogenous H_2_S produced by the colonic tissue devoid of mucosa and submucosa was significantly decreased in the WAS rats. Second, the expression of CSE and CBS in the colon devoid of mucosa and submucosa were both down-regulated in the WAS rats. Third, alternative blocking H_2_S-producing enzymes using PAG or AOAA, which mimiced a down “regulation” of the enzymes, produced a increase in the contractile activity in the SWAS rats. Because of the ‘nitric oxide (NO)-like effects’ [26], we did not test hydroxylamine (HA), the other inhibitor of CBS on the contractile activity in the SWAS rats. These results suggest an important role for the decreased H_2_S production in chronic WAS-induced colonic hypermotility.

In the present study, we indentified the localisation of H_2_S-producing enzymes. In both SWAS and WAS colon, CSE was strongly expressed in smooth muscle cells of the lamina propia whereas CBS-IR was less intense but still positive in muscle layers, which is consistent with the previous reports [26], [29]. In addition, we found the two H_2_S-producing enzymes were present in neurons of the myenteric plexus, and a similar staining was described in rat jejunum [30]. CSE-IR was intense in the nucleus of the myenteric neurons, whereas CBS-IR was weak in the cytosols. CSE appears to be the major source of H_2_S synthesis in both SWAS and WAS colon and CBS makes a minor contribution as well. Note that major differences might exist in rat colon. For example, CBS-IR was apparent in lamina propria but CSE-IR was quite diffuse [31], [32]. CSE was expressed in neurons of the enteric nervous system but CBS was not [26]. Perhaps the different technical approaches account for these major differences in the distribution of the enzymes.

Myenteric neurons, located between the longitudinal and circular muscle layers in the gastrointestinal wall, mainly regulate gastrointestinal motility [33]. CSE-IR or CBS-IR neurons, which can produce H_2_S, could be regarded as inhibitory motor neurons and therefore, H_2_S of neural origin can be considered a ‘neurotransmitter’. However, the release of H_2_S after neuronal stimulation was not demonstrated in enteric neurons [13] and H_2_S does not participate in neurally mediated relaxation [26]. Perhaps the appropriate parameters of stimulation have not been used to elicit H_2_S release [26]. Further studies are needed to evaluate the role of H_2_S of neural origin. Endogenous H_2_S of non-neural origin can be produced by smooth muscle cells. It may regulate motility through direct interaction with some molecular targets in the smooth muscle cells and might be an intracellular signaling molecule in this sense. The present study showed that CSE and CBS expression in the colon devoid of mucosa and submucosa were both down-regulated in the WAS rats. Theoretically, the decreased H_2_S production from either a muscular or a neuronal source might contribute to colonic motility disorder.

Several plausible mechanisms for colonic hypermotility were considered in chronic stress animal models. Central structures involved in stress-induced hypermotility include the increased mRNA expression of arginine vasopressin and corticotropin-releasing factor in the hypothalamus [5], [6]. We reported recently that the plasma hormones levels of substance P, thyrotropin-releasing hormone, motilin, and cholecystokinin in the WAS rats were increased, whereas peptide YY was decreased. These gastrointestinal hormones and the brain-gut peptides influenced colonic motility by directly decreasing the IKv and IBK_Ca_ of proximal colonic smooth muscle cells [2]. Moreover, chronic stress can remodel the gastrointestinal smooth muscle cells to cause motility dysfunction. For example, 9-day heterotypic chronic stress was found to increases the synthesis and release of norepinephrine in plasma, which enhanced the transcription of the pore-forming α_1C_ subunit of Ca_v_1.2 (L-type) channels in colonic circular smooth muscle cells [4]. And the cellular mechanisms by which norepinephrine enhances expression of the α_1C_–subunit is related to the phosphatidylinositol 3-kinase (PI3K)/Akt/GSK-3βsignaling pathway [7]. Indeed, H_2_S is intermingled with the synthetic and release pathway for some neural and or hormonal factors. For example, H_2_S can regulate the release of corticotrophin-releasing hormone and NO [28], [34], and enhance the ability of NO to relax smooth muscle [35]. On the other hand, NO can increas the expression and activity of CSE [28], [36]. Future studies should address the possible mechanisms behind chronic stress-induced H_2_S regulation in the colonic motility, and the link between H_2_S and neural and or hormonal factors in these changes.

NaHS, the H_2_S donor, is an important pharmacological tool for investigating the effects of H_2_S in vitro. Our data showed exogenously applied NaHS concentration-dependently inhibited the spontaneous motility of colonic muscle strips from both SWAS and WAS rats, which is consistent with the previous studies [13], [14], [15], [16], [17]. The inhibitory effect of NaHS on smooth muscle cells is largely through an direct action on K_ATP_ channels [9], [10], [14], [15], [18]. The result that glybenclamide, a K_ATP_ channel blocker, significantly reduced the inhibitory effect induced by NaHS raised the possibility that NaHS might act via K_ATP_ channel. The molecular mechanisms underlying the effect of H_2_S on K_ATP_ channels are still largely unknown. Because H_2_S is a reductant, it is possible that H_2_S directly interacts with K_ATP_ channel proteins by reducing the cysteine residues [37]. Taken together, less H_2_S production through “less activation” of K_ATP_ channels may be one of the reasons why repeated WAS induced colonic hypermotility.

It is well recognized that Kir 6.1/SUR2B and Kir 6.2/SUR2B form the K_ATP_ complex located in the GI smooth muscle cells [21], [22], [23], [24]. Heterologously expressed Kir 6.1 demonstrates a conductance of ∼35 pS, whereas Kir 6.2 has a conductance of ∼80 pS [21]. The data that the single-channel conductance of K_ATP_ channel is ∼42 pS and Kir 6.1 is strongly expressed on the plasma membrane, whereas Kir 6.2 appeared to be restricted to the cytosol, suggest that Kir 6.1 is the major pore-forming isoform of K_ATP_ channel [21]. Despite the observation of a marked decrease in the capacity of WAS colon to synthesize H_2_S, the pharmacological evidence in vitro showed that the NaHS IC_50_ for the WAS rats was significantly lower than that for the controls. This indicates either an increase in the number of K_ATP_ channels, or an increase in the frequency of openings in this model of WAS. We next investigated the expression of the isoforms of K_ATP_ channels. After WAS, the protein expression of the pore-forming Kir 6.1, except for Kir 6.2, and SUR2B, the major binding site for the K^+^ channel opener was markedly increased, indicating that the number of K_ATP_ channels was increased. We do not know the detailed mechanisms by which WAS enhances expression of Kir 6.1/SUR2B. As activation of K_ATP_ channels leads to the relaxation of GI smooth muscle [10], it may be an adaptive responses following exposure to a repeated psychological stressor in rats, aiming at improving disturbed gut function and ameliorating symptoms.

There is growing evidence that H_2_S might have important therapeutic potentia. For example, H_2_S maintains the gastric mucosal integrity and administration of H_2_S donor or L-cysteine, a precursor of endogenous H_2_S synthesis, results in enhanced gastric ulcer healing [28]. Moreover, H_2_S is an anti-inammatory mediator and promotes resolution of colitis in rats [32], [38]. Our pharmacological experiments in vitro showed that the IC_50_ values of NaHS for the WAS rats were lower than those for the controls, partly due to the increased expression of Kir 6.1 and SUR2B in smooth muscle cells. It is thus noteworthy that the H_2_S donor has potential contribution to reverse stress-induced colonic hypermotility. However, the putative beneficial vs toxic effects of H_2_S has been controversial for many years [26], [32]. Further studies are needed to evaluate its physiological, pharmacological and toxicological effects in GI tract.

In summary, this study provides evidence that the decreased production of endogenous H_2_S may be one of the reasons for chronic WAS-induced colonic hypermotility, and the increased expression of Kir 6.1 and SUR2B in colonic smooth muscle cells may be a defensive response to chronic stress. H_2_S donor can markedly inhibited the colonic contractions of the WAS rats. Thus, enhancement of endogenous H_2_S synthesis or delivery of appropriate concentrations of exdogenous H_2_S may have potential clinical utility in treating chronic stress-induced colonic hypermotility.
